# Gait analysis after total hip arthroplasty using direct anterior approach versus anterolateral approach: a systematic review and meta-analysis

**DOI:** 10.1186/s12891-019-2450-2

**Published:** 2019-02-08

**Authors:** Jun-Il Yoo, Yong-Han Cha, Kap-Jung Kim, Ha-Yong Kim, Won-Sik Choy, Sun-Chul Hwang

**Affiliations:** 1 Department of Orthopaedic Surgery, Gyeongsang National University Hospital, Jinju, South Korea; 2 Department of Orthopedic Surgery, Eulji University Hospital, Dunsan-Seoro 95, Seo-gu, Daejeon, 35233 South Korea

**Keywords:** Total hip arthroplasty, Direct anterior, Anterolateral, Harding, Gait analysis

## Abstract

**Background:**

Comparative studies of total hip arthroplasty using the direct anterior approach (DAA) compared with the anterolateral approach (ALA) by gait analysis compared the results of the two groups, the damage to the abductor muscle, with objective and detailed kinematic as well as kinetic data of actual gait. The purpose of this systematic review was to analyze the differences in gait such as time-dependent parameters, kinetics, and kinematics after THA using the DAA compared with ALA.

**Methods:**

PubMed Central, OVID Medline, Cochrane Collaboration Library, Web of Science, EMBASE and AHRQ carried out a comprehensive search for all relevant randomized controlled trials and comparative studies, up to December 2018. Based on the following criteria, studies were selected: 1) study design: randomized controlled trials or non-randomized comparative studies; 2) study population: patients with primary osteoarthritis or avascular necrosis; 3) intervention: total hip arthroplasty by DAA or ALA; 4) Kinetic and kinematic data after gait analysis in the plains during postoperative follow-up.

**Results:**

Of the 148 studies, 7 randomized controlled trials and 5 comparative studies were finally included in this systematic review. The peak hip flexion within 3 months after surgery was described in two studies and was significantly higher in the DAA group. (OR = 1.90; 95% CI [1.67,2.13]; *P* < 0.01, Z = 16.18). The gait speed within 3 months after surgery was reported in 3 studies and was significantly higher in the DAA group than in the ALA group. (SMD = 0.17; 95% CI [0.12,0.22]; *P* < 0.01, Z = 6.62) There was no difference between the two groups in stride length, step length, and hip range of motion in sagittal plane.

**Conclusions:**

In this meta-analysis, gait speed and peak hip flexion within 3 months after surgery were significantly higher in the DAA group than in the ALA group. Despite a few significant differences between two approaches, determining whether the reported differences in terms of postoperative gait values are clinically meaningful remains a substantial challenge.

**Electronic supplementary material:**

The online version of this article (10.1186/s12891-019-2450-2) contains supplementary material, which is available to authorized users.

## Background

Total hip arthroplasty (THA) reduces pain, and improves function and gait in patients with trauma or degenerative disease involving the hip joint [1]. The implant survival or patient-reported outcomes after THA via various surgical approaches yielded excellent results [2]. However, the survival of patients with the prosthesis is related to the type of bearing surface or the implant position. Therefore, the objective function of the patient cannot be determined using these assessment methods. In addition, the functional indicators of scores used in post-operative functional assessment do not adequately assess the functional status of the joint exercises actually used by the patient [3]. In this regard, gait analysis is a useful tool for the assessment of postoperative objective function after THA [4].

The direct anterior approach (DAA), which is used in THA, facilitates reaching the hip joint via intermuscular plane of the gluteus medius and sartorius. This surgical approach does not result in direct muscular damage and facilitates rapid recovery and early ambulation. [5]. It is technically demanding, however, with its own unique set of complications, which implies a significant period of learning [6]. Among the other approaches, an anterolateral approach (modified Harding, ALA) was performed to detach the gluteus medius and anterior one-third of the minimus to reach the hip joint and repair the muscle detached after insertion of the prosthesis [7]. Although the results are rapid and long-term follow-up is good, gait delay may occur due to muscle detachment and long-term functional challenges due to scar tissue formation or fatty degeneration [8, 9].

Comparative studies of THA results using both approaches reported the timing of gait, Harris hip score, complication rate, and radiological parameters [10]. These studies using gait analysis compared the results of the two groups, the damage to the abductor muscle, with objective and detailed kinematic as well as kinetic data of actual gait [7, 11, 12].

Therefore, the purpose of this systematic review was to analyze the differences in gait biomechanics such as time-dependent parameters, kinetics, and kinematics after THA using the DAA compared with the ALA.

## Methods

Our current review and meta-analysis were performed according to the Preferred Reporting Items for Systematic Reviews and Meta-Analyses (PRISMA) guidelines [13].

### Study eligibility criteria

Studies were selected on the basis of the following criteria: 1) study design: randomized controlled trials or non-randomized comparative studies; 2) study population: patients with primary osteoarthritis or avascular necrosis; 3) intervention: total hip arthroplasty by DAA or ALA; 4) kinetic and kinematic data after gait analysis in the plains during postoperative follow-up.

Studies were excluded if 1) they failed to meet the above criteria; 2) patients were affected by orthopedic, neurological or other disease affecting gait pattern; and 3) gait analysis was performed using stairs.

### Search methods for identification of studies

PubMed Central, OVID Medline, Cochrane Collaboration Library, Web of Science, EMBASE and AHRQ carried out a comprehensive search for all relevant randomized controlled trials and comparative studies, up to December 2018. We used the following search terms: “gait total hip approach”. A manual search of possibly related references was also conducted.(Additional file 1) Two researchers reviewed the titles, abstracts and full texts of all potentially relevant studies independently, as recommended by the Cochrane Collaboration [14]. Any disagreement was resolved by the third reviewer. Full-text articles of the remaining studies were assessed according to the previously defined inclusion and exclusion criteria, and then eligible articles were selected. The review authors were not blinded to authors, institutions, or the publication.

### Data extraction

The following data were extracted from the articles included: authors, date of publication, design of the study, participant features, follow-up period, specific interventions and outcome measurements. The outcomes pooled in this analysis included time distance parameters (gait speed, cadence, step length, step width, swing time), kinematics (hip joint range of motion, peak flexion or extension angle of hip joint during gait), and kinetics (hip moment). Kinetics was investigated but meta-analysis was not performed in this study because we did not get the data that can be used for statistical processing.

### Methodological quality assessment

Two authors independently evaluated the methodological quality of the included studies using the same criteria for RCTs as described in the Cochrane Manual for Systematic Intervention Reviews 5.2. The 10 criteria were: 1) allocation concealment; 2) clearly defined inclusion and exclusion criteria; 3) the results of withdrawn or excluded patients after allocation were described and included in the intention to treatment analysis; 4) groups well matched, or with appropriate covariate adjustment; 5) surgeons’ experience; 6) identical care programs other than the trial options; 7) clearly defined outcome measures in the text with a definition of any ambiguous terms encountered; 8) blinding of outcome assessors to assignment status; 9) appropriate timing of outcome measures; and 10) reported follow-up loss less than 5 % of participants.

The scale between Newcastle and Ottawa was used to evaluate the methodological quality of non- randomized studies. It consists of eight items classified into three dimensions: the selection of the study population, the comparability of the groups and the determination of exposure (case-control study) or outcome (cohort study). Each dimension consists of subcategorized questions: selection (up to 4 stars), comparability (up to 2 stars) and exposure (up to 3 stars). A study can therefore be awarded up to 9 stars with the highest quality. The quality of all the studies was assessed independently by two authors.

### Data analysis

In the included studies, the timing of gait analysis after total hip arthroplasty varied widely. So, we performed meta-analysis for the gait analysis performed within 3 months after THA to better clarify the differences between the two approaches. This meta-analysis was carried out with the software Review Manager (Rev Man 5.3) and the meaning was set to *P* < 0.05. For dichotomous results, the odds ratio (OR) and the confidence interval (CI) of 95% were calculated. For continuous outcomes, standardized mean difference (SMD) and CI was calculated at 95%. The heterogeneity size of the studies was estimated using I2 statistics and the Chi square test. A P of > 0.10 and an I^2^ of 50% were considered lacking statistical heterogeneity. Higgins I^2^ statistics was performed to test heterogeneity [15]. Significant heterogeneity was observed in these studies, and therefore, random effects or fixed effect models were adopted depending on the heterogeneity of the included studies. Sensitivity analysis was conducted by omitting a single study each time and building data from the remaining studies to explore possible high heterogeneity and to determine outcome stability.

## Results

### Search results

**Fig. 1:**
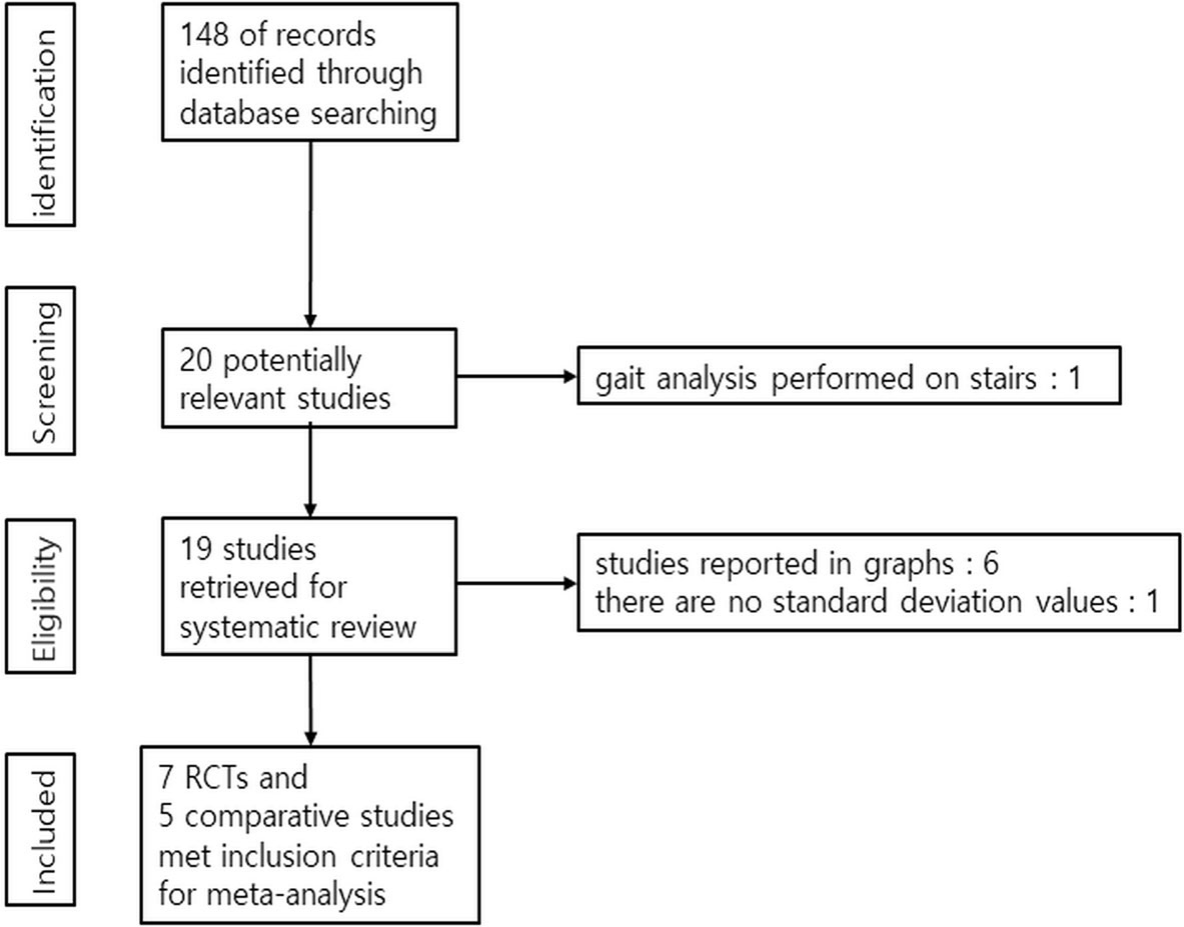
Preferred Reporting Items for Systemic Reviews (PRISMA) flow diagram outlining the clinical study selection process

The initial search returned 148 references from the selected databases. The selection of abstracts and titles for duplicates, unrelated articles, case reports, systematic reviews and non- comparative studies excluded one hundred and twenty- eight references. The remaining 20 studies underwent full-text review. A further 3 studies were excluded. The details of the relevant studies can be found in the study selection process flow chart (Fig. 1). Seven randomized controlled studies and 5 comparative retrospective studies, including 429 patients (211 from DAA group, 218 from ALA group), were finally selected [1, 8, 12, 16–24]. The main features and results of the meta-analyzed studies are shown in Table 1.

**Table 1:** Characteristics of the included studies

Study	type	DAA group (N)	ALA group (N)	Evaluation time	Variables
Mayr 2009 [16]	RCT	16	17	Preop, POD 6 W, 12 W	Time-distance parameters, pelvic & hip kinetic and kinematic parameters
Lugade 2010 [17]	CS	12	11	Preop, POD 6 W, 16 W	Symmetry index of time-distance parameters
Wesseling 2016 [23]	CS	23	8	After 1 year	Time-distance parameters, hip kinetics & kinematic parameters
Varin 2013 [24]	CS	20	20	After 10 months	Time-distance parameters, Hip, knee, ankle kinetic and kinematic parameters
Pospischill 2010 [18]	RCT	20	20	POD 10 days, 12 W	Time-distance parameters, pelvic and hip kinetic and kinematics parameters, electromyographical evaluation
Paliery 2011 [22]	RCT	15	15	POD 4 W, 13 W	Time-distance parameters, pelvic and hip kinetic and kinematic parameters, electromyographical evaluation, functional hip score
Muller 2012 [19]	RCT	15	15	Preop, POD 12 W	Time-distance parameters, foot progression angle
Queen 2011 [1]	RCT	15	8	Preop, POD 6 W	Time-distance parameters, hip kinetic and kinematic parameters, functional hip score
Klausmeier 2010 [8]	CS	12	11	Preop, POD 6 W, 16 W	Time-distance parameters, hip kinetic and kinematic parameters, functional hip score
Queen 2014 [12]	RCT	11	12	POD 1 year	Time-distance parameters, hip kinetic and kinematic parameters
Martin 2011 [20]	CS	42	41	POD 1 year	Time-distance parameters, hip kinetic and kinematic parameters, functional hip score
Kiss 2012 [21]	RCT	40	40	Preop, POD 12 W, POD 6 M, POD 1 year	Time-distance parameters, pelvis, hip, knee kinetic and kinematic parameters, functional hip score

*DAA* direct anterior approach, *ALA* anterolateral approach, *RCT* randomized controlled trial, *preop* preoperative, *POD* postoperative day, *W* weeks, *CS* comparative study

### Gait speed

**Fig. 2:**
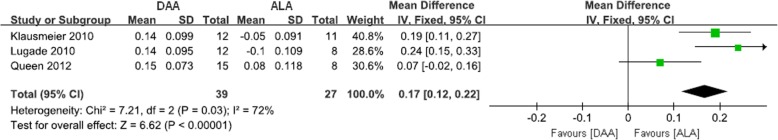
The forest plot of gait speed comparing DAA with ALA

Three articles reported gait speed in the gait analysis performed within the postoperative 3 months [1, 8, 17]. A total of 66 patients were enrolled, including 39 patients in the DAA group and 27 patients in the ALA. A high degree of heterogeneity was observed across studies (I^2^ = 72, *P* = 0.03). Therefore, the random effects model was used for data analysis. A statistically significant higher gait speed was observed in the DAA group than in the ALA group (OR: 0.17, 95% CI: 0.12 to 0.22, *P* < 0.01, Z = 6.62) (Fig. 2).

### Step length

**Fig. 3:**
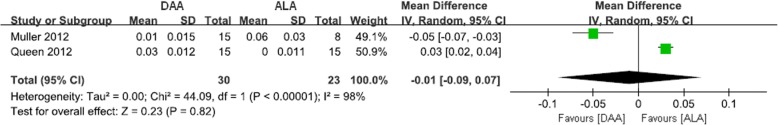
The forest plot of step length comparing DAA with ALA

Two studies performed gait analysis within 3 months postoperatively [1, 19]. A total of 53 patients were enrolled in the studies, including 30 patients in the DAA group and 23 patients in the ALA group. A high degree of heterogeneity was observed across the studies (I^2^ = 98, *P* < 0.01). Therefore, random effects model was used for data analysis. There was no statistically significant difference in step length within 3 months after THA between the two groups (OR: -0.01, 95% CI: -0.09 to 0.07, *P* = 0.82, Z = 0.23) (Fig. 3).

### Stride length

**Fig. 4:**
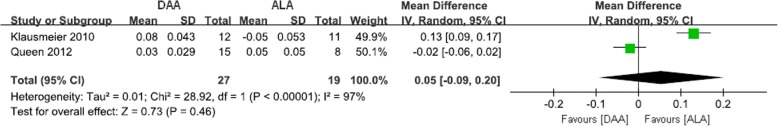
The forest plot of the stride length comparing DAA with ALA

Two studies reported stride length in gait analysis at 6 weeks postoperatively [1, 8]. A total of 46 patients were included, 27 in the DAA group and 19 in the ALA group. Significant heterogeneity was observed across studies (I^2^ = 97, *P* < 0.01). Therefore, a random effects model was used for data analysis. No statistically significant difference was evident in stride length within 6 weeks after THA between the two groups (OR: 0.05, 95% CI: -0.09 to 0.20, *P* = 0.46, Z = 0.73) (Fig. 4).

### Peak hip flexion

**Fig. 5:**
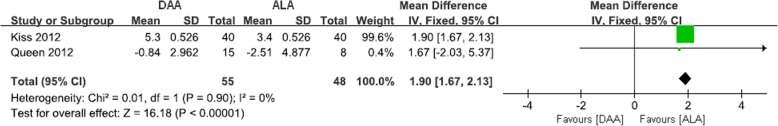
The forest plot of peak hip flexion comparing DAA with ALA

Two studies reported peak hip flexion in gait analysis within 3 months postoperatively [1, 21]. A total of 103 patients were included, 55 in the DAA group and 47 in the ALA group. A low degree of heterogeneity was detected across studies (I^2^ = 0, *P* = 0.90). Therefore, a fixed effects model was used for data analysis. A statistically significant higher peak hip flexion was found in the DAA group than in the ALA group (OR: 1.90, 95% CI: 1.67 to 2.13, *P* < 0.01, Z = 16.18) (Fig. 5).

### Hip range of motion (ROM) in sagittal plane

**Fig. 6:**
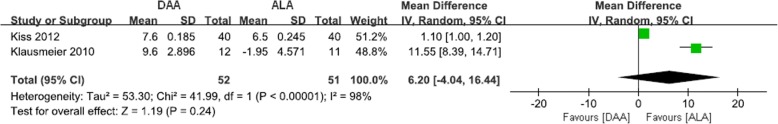
The forest plot of the hip range of motion (ROM) in sagittal plane comparing DAA with ALA

Two studies reported hip ROM in sagittal plane during gait analysis within 3 months postoperatively [8, 21]. A total of 103 patients were included, 52 in the DAA group and 41 in the ALA group. Significant heterogeneity was observed across studies (I^2^ = 98, *P* < 0.01). Therefore, a random effects model was used for data analysis. There was no statistically significant difference in hip ROM in sagittal plane within 3 months after THA between the two groups (OR: 6.20, 95% CI: -4.04 to 16.44, *P* = 0.24, Z = 1.19) (Fig. 6).

## Discussion

In this meta-analysis, gait speed and peak hip flexion within 3 months after surgery were significantly higher in the DAA group than in the ALA group, and there was no difference between the two groups in stride length, step length, and hip range of motion in sagittal plane.

### Time distance parameter

During gait analyses within 16 weeks after operation, the time-distance parameters varied between the two groups (Table 2). Mayr and Klausmeier reported that in the gait analysis performed at 6 weeks postoperatively, the time-distance parameters were better in the DAA group [8, 16]. In addition, Mayr reported that the DAA group showed increased cadence, stride time and length, and walking speed at 12 weeks postoperatively, without any changes observed in the ALA group. Klausmeier reported that the time-distance parameters improved at 16 weeks postoperatively than at 6 weeks in both groups, although a significantly higher improvement was observed in the DAA group. However, in a study by Queen, Popsppischill, and Muller, gait analysis within 16 weeks after surgery showed no difference between the two groups, and Paliery reported that the swing phase of the affected limbs was significantly longer, without any significant differences in the remaining time-distance parameters [1, 18, 19, 22]. There was, however, no difference in time-distance parameters between the two groups during gait analysis after more than 10 months [12, 17, 22, 23]. In our meta-analysis, the gait speed within 3 months after surgery was superior to ALA group than DAA group. This suggests that the recovery of ambulatory function occurred much earlier in the DAA group than in the ALA group. However, as Varin et al. stated the time-distance parameters are not direct indicators of joint coordination, moment production, and force distribution, gait speed do not represent a direct function of the hip joint. Therefore, we should be careful when interpreting this result [24].

**Table 2:** Main findings of time distance parameters in the included studies

Study	Main findings
Mayr 2009 [16]	At 6 weeks postoperatively, the rate of single support of the operated limb increased in the DAA group and at 12 weeks postoperatively, the cadence, stride time and length, and walking speed increased significantly. However, no increase was observed in the ALA group at 6 and 12 weeks after surgery.
Klausmeier 2010 [8]	At 6 weeks postoperatively, single limb support time symmetry improved in the DAA group, but not in the ALA group. Gait velocity and step width were not different between the two groups. At 16 weeks postoperatively, both groups showed improved single limb support time symmetry and gait velocity. The degree of improvement was higher in the DAA group and the step length symmetry in the DAA group also improved
Queen 2011 [1]	There was no statistically significant difference in stance time and swing time between the two groups.
Pospischill 2010 [18]	There was no difference in the time-distance parameters between the two groups at 10 and 12 weeks postoperatively.
Paliery 2011 [22]	The swing phase of the affected limb was significantly longer in the DAA group at 30 days postoperatively, but the other time-distance parameters did not differ between the two groups.
Muller 2012 [19]	There was no difference in gait velocity, cadence, step length, and stance duration between two groups at 3 months postoperatively.
Varin 2013 [24]	At 10 months postoperatively, no differences were found in the time-distance parameters between the two groups compared with the control group.
Martin 2011 [20]	No statistically significant difference was found in cadence and gait velocity between the two groups at 1 year after surgery.
Kiss 2012 [21]	No difference in time-distance parameters was found between the two groups at 1 year postoperatively
Queen 2014 [12]	No difference in stance time, swing time, and step length was detected between two groups at 1 year after surgery.

*DAA* direct anterior approach, *ALA* anterolateral approach

### Hip kinetics and kinematics

In the short and long-term follow-up of gait analysis, the results of hip joint kinetics and kinematics were interpreted in various ways (Table 3). Mayr and Lugade et al. found a disparity in terms of gait analysis between the two groups, and DAA group showed faster recovery, and Kiss reported that Wesseling study gait analysis performed a year after operation revealed significantly greater improvement in gait in ALA close to normal gait and resulted in better functional outcome than in DAA group [16, 17, 21]. In our meta-analysis, the peak hip flexion within 3 months was significantly greater in the DAA group than in the ALA group. However, in the remaining studies, there was no difference in the results of gait analysis between the two groups, although a few data showed differences. However, caution is needed when interpreting the results of gait analysis in the above studies. Gait utilizes all the joints and muscles from the pelvis to the ankle, and therefore challenges associated with gait can be detected directly in the hip joint, although it can be observed in the form of compensation movement in the hip or other joints. The authors also believed that these differences were due to various forms of damage to the abductor, resulting in the difference between the two approaches.

**Table 3:** Kinetics and kinematics of included studies

Study	Evaluation time	Differences between the two groups		Results
		DAA group	ALA group	
Mayr 2009 [16]	POD 6 week	Hip flexion, extension range increased	Maximum internal rotation of the hip in stance increased exclusively	DAA resulted in faster recovery of function at 6 weeks and 12 weeks postoperatively.
	POD 12 week	Hip flexion at foot contact, maximum flexion in swing, ROM in sagittal and coronal planes, maximum adduction in stance	Hip flexion in foot contact, maximum flexion in swing, ROM in the transverse plane, maximum internal rotation in stance	
Lugade 2010 [17]	POD 6 W, 16 W		Greater asymmetry in the ALA group compared with DAA group.	Both groups recovered gait symmetry at 16 weeks postoperatively, but DAA group recovered faster than DAA group at 6 weeks postoperatively.
Pospischill 2010 [18]	POD 10 days, 12 week	Despite absence of statistical significance, hip extension range decreased more than in ALA group		No significant difference between the two groups; Patients after hip surgery adjusted their gait pattern to reduce the magnitude of loading on hip joint.
Queen 2011 [1]	POD 6 week			No difference in hip flexion at heel strike, peak hip flexion, peak hip abduction angle, or peak vertical ground reaction force
Muller 2012 [19]	POD 12 week			No effect of the surgical approach on the gait patterns or foot progression angle
Klausmeier 2010 [8]	POD 16 week	Better peak external rotation moment		No significant difference between the two groups
Paliery 2011 [22]	POD 4 week	Longer duration of swing phase, improved range of motion of hip, reduced adduction		Gait pattern after THA strictly dependent on the surgical access and mainly on the extent and location of the surgical damage.
	POD 13 week	Better hip flexion, minor obliquity of pelvis	Better hip extension	
Varin 2013 [24]	POD 10 months	Peak hip abduction moment reduced more than ALA group		DAA group showed closer to normal sagittal plane kinematics at the hip than the ALA group. Factors other than surgical approach contributed more to difference between the two groups.
Wesseling 2016 [23]	POD 1 year	Hip Abduction moment reduced more than ALA group	Hip flexion moment reduced more than DAA group	No significant difference between the two groups; Patients after hip surgery adjusted their motion pattern to decrease the magnitude of loading on the hip joint.
Queen 2014 [12]	POD 1 year	Decreased operative hip adduction moment	Increased adduction moment	No significant difference between the two groups
Martin 2011 [20]	POD 1 year	Heel interval during walking was greater with DAA group		Difference in terms of unoperated leg, while there is no difference between the two groups
Kiss 2012 [21]	POD 1 year	Gait pattern was almost normal	Hip motion reduced, pelvic rotation increased, increased compensation of knee and hip motion of unoperated leg	DAA yielded better functional outcome compared with ALA

*POD* postoperative day, *ROM* range of motion, *DAA* direct anterior approach, *ALA* anterolateral approach, *THA* total hip arthroplasty

### Soft tissue injury

In the Wesseling study, there was a difference in hip flexion moment and abduction moment between the two groups [23]. In this study, no significant difference in loading was detected in the hip joint after one year of operation, but the hip flexion moment was lower in the ALA group than in the DAA group, and the hip abduction moment was lower in the DAA than in the ALA group. Meneghini et al. reported that 2.6% of the muscular area of the gluteus medius, 8.5% of the muscular area of the gluteus minimus, and 31.3% of the muscular area of the tensor fascia lata (TFL) were damaged during DAA in the cadaveric study [25]. Wesseling et al. asserted that gluteus minimus and medius are the main abductors of hip, and TFL is also a major hip abductor and DAA-induced TFL injury results in a decrease in hip abduction moment in the DAA group [23].

However, Varin et al. suggested that the peak hip abduction moment in the gait analysis at 10 months postoperatively was lower in DAA than in control or ALA, which may be attributed to female dominance [24]. In Palieri’s study, the gait analysis of the ALA group showed a decrease in hip flexion and abnormal EMG findings postoperatively at 10 days, suggesting that the anterior 1/3 of abductor muscle played a major role in hip flexion [22]. The reduction in hip flexion moment in the ALA group reflected abductor damage. In the Wesseling and Pospischill study, TFL injury in the DAA group showed that the degree of traction injury during surgery varied depending on the operator’s experience or skill [18, 23]. In Martin’s study, sufficient exposure of the femoral shaft through the DAA was considered a technical challenge [20]. Excessive tension on the posterior femoral neck by the Hohmann retractor may result in damage to the fibers of the gluteus medius around the insertion of the greater trochanter.

### Gait mechanism of ALA group different from DAA group

In the systematic review, many studies have reported that ALA group shows different gait mechanisms compared to DAA group, which is thought to be caused by the difference in approach.

Pospischill et al. reported that one of the ALA group patients developed Trendelenburg gait 10 days postoperatively and disappeared around 12 weeks postoperatively [18]. In this study, 5 changes in the DAA group and 6 changes in the ALA group occurred. However, no change in the EMG of the TFL was detected in the DAA, although 2 statistically significant changes were found in the ALA group suggesting that ALA damages the abductor and TFL, and Trendelenburg gait was a compensatory mechanism to reduce hip joint loading. However, Pospischill et al. suggested possible soft tissue sparing with ALA because electromyogram (EMG) recovery was observed around 12 weeks [18].

Varin et al. also found that the pelvic ROM of the ALA group was 7.7 ° ± 2.4 °, which was not significantly different from the normal group; however, DAA presented with pelvic obliquity (8.4 ° ± 2.5 °) closer to normal range, which suggested an impact of the damaged abductor even though it failed to result in Trendelenburg gait [24]. By contrast, Mayr et al. reported that pelvic rotation in coronal plane and hip adduction in stance indicated abductor weakness; however, no significant changes in ALA group was observed at 6 and 12 weeks compared with DAA group [16]. Klausmeier et al. reported no changes in pelvic obliquity between the two groups at 6 weeks postoperatively [8]. However, these results may not be statistically significant due to the subtle change in pelvic obliquity and the lack of evaluation of compensation based on trunk kinematics. However, in the Palieri’s EMG analysis, chaotic timing of activation of gluteus medius was observed in the ALA group at 30 days postoperatively, and further hip flexion and minimal pelvic obliquity were observed in the ALA group at 90 days postoperatively, suggesting that recovery of the gluteus repair site after surgery may affect pelvic obliquity or hip flexion pattern [22].

The hip adduction moment increased in the ALA group. In the study by Queen et al., the ALA group exhibited a higher adduction moment compared with the unoperated hip, while a decreased hip adduction moment was observed in the DAA group compared with the unoperated hip. [12] Such outcome is probably due to the relative increase in adductor moment at the operative hip in patients included in the ALA group, which may have corresponded with pelvic drop during the propulsion phase. It may be attributed to weak abductor mechanism.

The internal rotation of hip joint and internal rotation of foot progression angle correlated with reduced abductor function. Abductor moment arm is compensated by internal rotation of hip in patients with reduced abductor function [26]. In the study by Mayr, the ALA group presented with increased maximum internal rotation of the hip in stance postoperatively after 6 weeks, and the increased hip flexion and ROM in the transverse plane, maximum internal rotation in stance at 12 weeks postoperatively, which was not observed in the DAA group [16]. This finding assumed that fatty atrophy of the anterior 1/3 of the gluteus medius by its anterior detachment leads to functional impairment and compensates for such impairment in ALA group. Muller et al. also state that anterior fiber of gluteus medius muscle also contributes to internal rotation and posterior fiber contributes to external rotation [9]. Therefore, the injury to the muscle of the abductor during ALA increased the external rotation of the lower limb, including the foot, in patients with complete gluteal insufficiency in the supine position. However, Muller et al. found a lack of significant difference in foot progression angle between DAA and ALA due to hypertrophy of TFL [27]. Martin et al. stated that ALA group was presented with internal rotation of foot progress angle compared with DAA group a year after operation and probably because femoral anteversion was not adequately evaluated [20].

Increased pelvic or knee motion occurred in the ALA group. Gait analysis during a year after operation showed that DAA group presented with gait pattern similar to the control group compared with ALA group. The study by Kiss showed that decreased hip motion in ALA group was compensated by another joint in the kinetic chain including knee motion and pelvic obliquity [21]. Increased pelvic obliquity and flexion-extension compensated for the decreased hip motion during the earlier post-operative period in ALA group, and was similar to the report by Miki et al. [28]. Huang et al. and Wu et al. also stated that pelvic rotation played an important role in increasing the step length during limited hip motion [29, 30]. In the study by Kiss, during the first postoperative month, the ALA group was unable to increase pelvic rotation because the gluteus medius and the posterior capsule were affected during surgery via ALA [21]. The significant decrease in step length of ALA group in the early postoperative months may be attributed to decreased hip motion and pelvic rotation. It can be hypothesized that in the late postoperative period the normal function of the gluteus medius and the posterior capsule was restored, facilitating increased pelvic rotation in the ALA group. Increased pelvic rotation resulted in step lengths that did not differ significantly from normal values despite decreased hip motion. Six months after surgery, the step length recovered to normal values with increased pelvic rotation, although hip motion was decreased.

The limitations of the study are as follows. First, this study lacked evaluation of preoperative mechanism of compensation for each study, which may have influenced postoperative gait analysis. In Klaumeier’s study, gait asymmetry in both groups at 16 weeks postoperatively was attributed to compensatory motion for osteoarthritis induced pain [8]. Petis’ study also referred to gait defect detected prior to operation [7]. Second, the recovery of strength and proprioception of injured muscle may vary with the different types of approach, and capsulotomy or capsulectomy affected the outcome [16, 31]. Third, the data of the included studies have heterogeneity. This is the limit of the meta-analysis, but we used a random model instead of a fixed model [32]. Forth, because the timing of gait analysis was varied, it did not include all the studies in each meta-analysis. Further studies will be needed in the future.

## Conclusion

In this meta-analysis, gait speed and peak hip flexion within 3 months after surgery were significantly higher in the DAA group than in the ALA group, and there was no difference between the two groups in stride length, step length, and hip range of motion in sagittal plane. Despite a few significant differences between two approaches, determining whether the reported differences in terms of postoperative gait values are clinically meaningful remains a substantial challenge.

## Additional file

Additional file 1: Detailed search strategies for each database. Mesh terms, search terms, and combinations of the two were used for each database search. (DOC 29 kb)

## Abbreviations

ALA: Anterolateral approach
CI: Confidence interval
DAA: Direct anterior approach
EMG: Electromyogram
OR: Odds ratio
RCTs: Randomized controlled trials
ROM: Range of motion
SMD: Standardized mean difference
THA: Total hip arthroplasty
