# Supplementary material for: Gait analysis after total hip arthroplasty using direct anterior approach versus anterolateral approach: a systematic review and meta-analysis
Source: BMC Musculoskelet Disord. 2019 Feb 8;20:63. doi: 10.1186/s12891-019-2450-2 (PMC6368707; doi:10.1186/s12891-019-2450-2)
Supplement: Supplementary file 1 — Detailed search strategies for each database. Mesh terms, search terms, and combinations of the two were used for each database search. (DOC 29 kb) [file 12891_2019_2450_MOESM1_ESM.doc]

Additional file 1. Detailed search strategies for each database. Mesh terms, search terms, and combinations of the two were used for each database search.

| Database | Detailed search strategies |
| --- | --- |
| MEDLINE/PUBMED | ("gait"[MeSH Terms] OR "gait"[All Fields]) AND total[All Fields] AND ("hip"[MeSH Terms] OR "hip"[All Fields]) AND approach[All Fields]) |
| EMBASE | 'gait total hip approach' OR (('gait'/exp OR gait) AND total AND ('hip'/exp OR hip) AND approach) |
| Cochrane Central Register of Controlled Trials | (gait) OR (hip AND total) OR (approach) |
| Web of Science | ((gait) AND (hip AND total AND (approach))) |
